# Obstetrical and Neonatal Outcomes in Twin Pregnancies Based on Chorionicity: A Systematic Review of ART-Conceived Monochorionic vs. Dichorionic Twins

**DOI:** 10.3390/jcm15124761

**Published:** 2026-06-18

**Authors:** Atieh Karimzadeh, Zahra Karimizadeh, Nazila Heidari, Samira Parviziomran, Sepehr Ramezanipour, Amirali Kalantari, Shahdad Farokhmanesh, Ibrahim Alkatout, Leila Allahqoli

**Affiliations:** 1 School of Medicine, Iran University of Medical Sciences, Tehran 1449614535, Iran; a.karimzadeh99@gmail.com (A.K.); nazila.heidari76@gmail.com (N.H.); 2 School of Medicine, Tehran University of Medical Sciences, Tehran 1416753955, Iran; zahra79kmzzz@gmail.com (Z.K.); samira.parvizi.omran@gmail.com (S.P.); amirali7980@gmail.com (A.K.); 3 Student Research Committee, Fasa University of Medical Sciences, Fasa 7461686688, Iran; sepehrramezanipoor@gmail.com; 4 Professor Alborzi Clinical Microbiology Research Center, Shiraz University of Medical Sciences, Shiraz 7193711351, Iran; shahdad.farokhmanesh81@gmail.com; 5 Department of Obstetrics and Gynecology, University Hospital Schleswig-Holstein, Campus Kiel, 24105 Kiel, Germany; ibrahim.alkatout@uksh.de; 6 Faculty of Health Sciences, Cyprus International University, 99258 Nicosia, Cyprus

**Keywords:** assisted reproductive technology, chorionicity, dichorionic twins, gestational age, monochorionic twins, neonatal outcomes, obstetrical outcomes, twin pregnancy

## Abstract

**Background:** Assisted reproductive technology (ART) is increasingly utilized worldwide, and approximately 30% of ART pregnancies result in twin gestations. Chorionicity strongly influences perinatal risk, yet its specific impact on ART-conceived twins has not been systematically clarified. **Objective**: To compare obstetrical and neonatal outcomes in assisted ART-conceived monochorionic (MC) versus dichorionic (DC) twin pregnancies and evaluate the impact of chorionicity on maternal and perinatal outcomes. **Methods:** This systematic review was conducted according to PRISMA guidelines and registered in PROSPERO (CRD42024600292). PubMed, Scopus, and Web of Science were searched through October 2024 for studies comparing obstetrical and neonatal outcomes in ART-conceived monochorionic and dichorionic twin pregnancies. Eligible studies were qualitatively synthesized. **Results:** Thirty-five studies comprising 15,648 ART-conceived twin pregnancies were included, including 371 monochorionic and 15,277 dichorionic pregnancies. MC pregnancies consistently demonstrated less favorable perinatal outcomes compared with DC pregnancies, including an earlier gestational age at delivery, increased prematurity, lower birth weight, and higher rates of perinatal mortality. By contrast, maternal complications, such as hypertensive disorders, gestational diabetes mellitus, PROM, and cesarean delivery, varied considerably across the studies without a consistent association with chorionicity. The baseline maternal characteristics were generally comparable between the groups. **Conclusions:** Monochorionicity in ART-conceived twin pregnancies is associated with increased adverse neonatal and perinatal outcomes, particularly prematurity and perinatal mortality, while maternal outcomes appear less clearly influenced by chorionicity. Standardized prospective studies are needed to further clarify the chorionicity-specific risks in ART twin pregnancies.

## 1. Introduction

For those battling infertility, assisted reproductive technology (ART) has become a ray of hope since Louise Joy Brown’s birth in 1978, which marked the first successful application of in vitro fertilization (IVF) [1,2,3].

Still a major public health concern, the worldwide prevalence of infertility greatly influences the personal, social, and financial spheres of the lives of the impacted people and families. According to studies, primary infertility affects about 45.85% of women and 51.5% of men [4]. In the United States, 8.5% of women between the ages of 15 and 49 are classified as infertile, while 13.4% suffer from reduced fecundity [5]. Globally, about 10–11% of women who are actively seeking pregnancy have undergone infertility treatments [6]. The use of ART has been steadily increasing. By 2019, ART accounted for 1.8% of all the births in the United States [7]. As of 2020, an estimated 8 million infants have been born through the use of ART, providing new opportunities for individuals and families navigating the challenges of infertility [8].

Defined by the American Center for Disease Control (CDC), ART is a spectrum of fertility treatments involving the manipulation of eggs or embryos. Procedures involving only the manipulation of sperm, such as intrauterine inseminations (IUI), should not be included under this definition. Furthermore, treatments involving ovarian stimulation (OS) without a clear intention for egg retrieval are also not classified as ART under this definition [9].

Although many infertile couples look to ART for help in conception, many studies have shown that the risks associated with this process are quite high. With almost 30% of ART pregnancies resulting in twins or higher-order multiple gestations, as opposed to 1–2% in natural conceptions, one major issue is the higher incidence of multiple pregnancies from ART procedures compared to natural conception. Furthermore, ART treatments are linked to complications such as ovarian hyperstimulation syndrome (OHSS), higher rates of gestational diabetes, hypertension, cesarean delivery (CD) [3,10], postpartum hemorrhage, and birth defects [11,12,13,14,15,16].

Among the pregnancies conceived through the use of ART, twin pregnancies are at an increased risk of maternal and neonatal morbidity and mortality compared to singleton pregnancies [17,18,19]. These pregnancies are characterized by a range of complications, including fetal anomalies, fetal death, intrauterine growth restriction, prematurity, polyhydramnios, and oligohydramnios [20]. Regardless of zygosity, monochorionic twin pregnancies are associated with even worse outcomes when compared to dichorionic twin pregnancies [21].

In this study, we aimed to investigate the adverse maternal and neonatal outcomes of ART-conceived twins and compare these outcomes based on chorionicity to gain a better understanding of ART twins, thereby highlighting the associated risk factors and improving pregnancy planning.

## 2. Materials and Methods

### 2.1. Protocol and Guideline

This study adhered to the Preferred Reporting Items for Systematic Reviews and Meta-Analyses (PRISMA) guidelines. The review protocol was prepared and registered with the International Prospective Register of Systematic Reviews (PROSPERO number: CRD42024600292) [22]. Ethical approval was deemed unnecessary for the conduct of this study. The PRISMA checklist is provided as Supplementary Table S1.

### 2.2. Inclusion Criteria

This systematic review encompassed all the studies focusing on twin pregnancies conceived through the use of ART, such as IVF, intracytoplasmic sperm injection (ICSI), pronuclear stage tubal transfer (PROST), gamete intrafallopian transfer (GIFT), and zygote intrafallopian transfer (ZIFT), that reported obstetrical and/or neonatal outcomes based on chorionicity. Studies exclusively involving sperm manipulation and treatments involving ovarian stimulation without a clear intent for egg retrieval were not considered as ART within the scope of this study.

### 2.3. Exclusion Criteria

This review excluded the following types of publications: case reports, case series, commentaries, brief communications, studies published in languages other than English, editorials, letters, and articles lacking full-text availability. Studies that did not align with the outcome of interest of this study were also excluded. Moreover, any studies that presented mixed data from ART and non-ART pregnancies, as well as those combining data from twins with singletons and/or other multiple pregnancies, vanishing twin syndrome, and fetal reduction were omitted [23]. Pregnancies that experienced selective fetal termination or multifetal pregnancy reduction were excluded from the analysis.

### 2.4. Outcomes

Focusing on the role of chorionicity, the main goal of this review was to compare the obstetrical and neonatal outcomes in pregnancies conceived through the use of ART.

In this review, obstetrical outcomes included premature rupture of membranes (PROM), placenta previa, placental abruption, postpartum hemorrhage, intrahepatic cholestasis of pregnancy (ICP), gestational hypertension (GHTN), preeclampsia, oligohydramnios, polyhydramnios, gestational diabetes mellitus (GDM), mean gestational age at delivery (weeks), preterm birth <37 weeks, and CD incidence.

Furthermore, neonatal outcomes included birth weight (in grams (gr)), intrauterine growth restriction (IUGR), low birth weight (LBW), small for gestational age (SGA), stillbirth, neonatal mortality, sex (male), 5 min Apgar < 7, hypoxic-ischemic encephalopathy (HIE), neonatal acidosis, respiratory distress syndrome (RDS), intraventricular hemorrhage (IVH), necrotizing enterocolitis (NEC), hyperbilirubinemia, neonatal sepsis, congenital malformations, twin to twin transfusion syndrome, and neonatal intensive care unit (NICU) admission and the duration of the NICU admission. The definitions from the original articles were adopted and accepted in this review (Supplementary Table S2).

### 2.5. Search Strategy

Three major electronic databases—PubMed, Scopus, and the ISI Web of Science—were searched for the relevant literature. In order to find studies on twin pregnancies conceived through the use of ART, the search strategy combined Medical Subject Headings (MeSH) with pertinent keywords. The search terms included the following: (“Twin Pregnancy”) AND (“Assisted Reproductive Technique” OR “In Vitro Fertilization” OR “Intracytoplasmic Sperm Injection” OR “Pronuclear Stage Tubal Transfer” OR “Gamete Intrafallopian Transfer” OR “Zygote Intrafallopian Transfer”) AND (“Maternal Outcome” OR “Gestational Age” OR “Pregnancy-Induced Hypertension” OR “Preeclampsia” OR “Oligohydramnios” OR “Polyhydramnios” OR “Gestational Diabetes Mellitus” OR “Premature Rupture of Membranes” OR “Placenta Previa” OR “Placental Abruption” OR “Postpartum Hemorrhage” OR “Intrahepatic Cholestasis of Pregnancy” OR “Cesarean Section”) AND (“Perinatal Outcomes” OR “Birth Weight” OR “Intrauterine Growth Restriction” OR “Low Birth Weight” OR “Small for Gestational Age” OR “Stillbirth” OR “Neonatal Mortality” OR “Apgar Score” OR “Hypoxic-Ischemic Encephalopathy” OR “Neonatal Acidosis” OR “Respiratory Distress Syndrome” OR “Intraventricular Hemorrhage” OR “Necrotizing Enterocolitis” OR “Hyperbilirubinemia” OR “Transient Tachypnea of the Newborn” OR “Twin-to-Twin Transfusion Syndrome” OR “Neonatal Intensive Care Unit Admission” OR “Neonatal Sepsis” OR “Congenital Malformations”). Only research released up until 10 October 2024 was included in the search. Every article that was retrieved was screened and assessed using predetermined inclusion and exclusion criteria.

### 2.6. Study Selection

**Figure 1:**
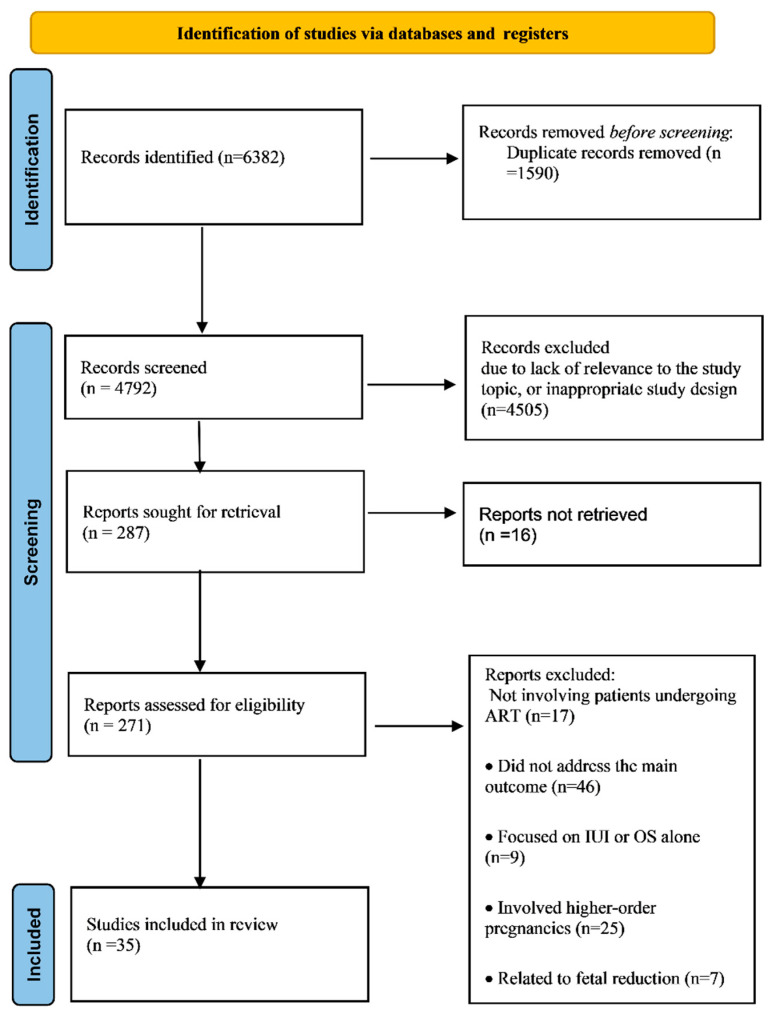
The study selection process. ART: assisted reproductive technology, IUI: intrauterine inseminations, OS: ovarian stimulation.

Following the removal of duplicate records, the screening process for determining study eligibility was conducted in two further stages. Three reviewers (S.P., N.H., and S.R.) first independently assessed the titles and abstracts of the retrieved articles to check for the relevance to the objectives of this study. A full-text evaluation of the chosen papers in the second phase was performed by three additional reviewers (L.A., Z.K., and A.K.) to ascertain their appropriateness for inclusion. During the selection process, disputes were resolved through discussions until all reviewers gave their approval. Every eligible study was critically assessed using a standardized data extraction form that covered key factors, such as study design, methodology, participant characteristics, intervention details, and reported obstetrical and neonatal outcomes. A PRISMA flow diagram was used to illustrate the selection process (Figure 1), offering a visual representation of the stages of study identification, screening, eligibility, and inclusion.

### 2.7. Data Synthesis and Extraction

Three reviewers (S.P., N.H., and S.R.) separately extracted data from the selected studies using a customized data collection table created using Microsoft Excel 2016. The extracted data were divided into the following domains:

1. Study demographics, including study title, name of the primary author, year of publication, country, and sample size.
2. Maternal characteristics, such as age distribution (years), body mass index (BMI) (kg/m^2^), and nulliparity status.
3. Obstetrical outcomes included gestational age at delivery (weeks), GHTN, preeclampsia, oligohydramnios, polyhydramnios, GDM, PROM, placenta previa, placental abruption, PPH, ICP, and CD.
4. Neonatal outcomes included birth weight (g), IUGR, LBW, SGA, stillbirth, neonatal mortality, Apgar scores, RDS, NICU admission, and congenital malformations.

All of the extracted information was systematically organized and presented in tabular format, providing a comprehensive summary of study characteristics and outcome data.

### 2.8. Assessment of Risk Bias

Two reviewers (L.A. and A.K.) independently evaluated the risk of bias using the Newcastle–Ottawa Scale for studies with two comparison groups and the NIH quality assessment tool for single-group studies [24].

## 3. Results

### 3.1. Search Results

A total of 6382 publications were found after a preliminary search across the databases; 1590 of these were deemed to be duplicates and were later eliminated. A total of 4505 studies were excluded as a result of title and abstract screening due to irrelevance to the study’s focus or an improper design. This resulted in 287 articles selected for full-text assessment; however, full-text versions were not accessible for 16 of these. Among the remaining 271 articles, further screening excluded 17 studies that did not involve patients undergoing assisted reproduction, 46 that did not address the main outcome of interest, 8 that focused on IUI or OS, 25 involving higher-order pregnancies, 7 concerning fetal reduction, and 127 that were not based on chorionicity. Of the 42 articles considered for potential inclusion, 7 did not provide enough details about whether IUI/OS or fetal reduction were part of the study process. The corresponding authors were contacted for clarification, and two responded: one confirmed the inclusion of IUI, and the other was included in the study [23]. After a full-text review and eligibility assessment, 35 studies met all the inclusion criteria and were included in the final qualitative synthesis [23,24,25,26,27,28,29,30,31,32,33,34,35,36,37,38,39,40,41,42,43,44,45,46,47,48,49,50,51,52,53,54,55,56,57,58] (Table 1).

### 3.2. Synthesis of Results

This systematic literature review analyzed 31 retrospective [23,25,26,27,28,29,30,31,32,33,34,35,36,37,38,39,41,42,43,44,45,46,47,48,49,50,51,52,54,56,57,58] and 4 prospective cohort studies [37,40,53,55] from 2004 to 2024. Based on the Newcastle–Ottawa Scale and NIH quality assessment tool [24], most studies were rated as moderate quality [23,25,26,27,28,30,31,33,37,38,39,40,41,42,43,44,45,46,47,50,51,52,53,54,55,56], while a smaller proportion were classified as good quality [29,34,35,36,48,49,57], and only one study [32] was rated as fair quality. The predominance of retrospective study designs and moderate methodological quality should be considered when interpreting the findings.

A total of three studies exclusively investigated monochorionic twin pregnancies [23,34,35], while five studies provided data on both monochorionic and dichorionic twins [25,26,29,32,38]. The remaining 27 studies focused solely on dichorionic twin pregnancies [28,29,30,31,32,33,34,35,36,37,38,39,40,41,42,43,44,45,46,47,48,49,50,51,52,53,54]. The dataset encompasses a total of 15,648 twin pregnancies, with 371 monochorionic pregnancies and 15,277 dichorionic pregnancies. Sample sizes varied significantly, ranging from 10 [23] to 164 [25] for monochorionic pregnancies and from 43 [56] to 6101 [25] for dichorionic pregnancies. Table 1 summarizes the characteristics of the included studies.

### 3.3. Maternal Characteristics and Obstetrical Outcomes

#### 3.3.1. Maternal Demographics

##### Maternal Age

Maternal age was consistently reported across the studies and showed remarkable similarity between the chorionicity groups, with the mean ages uniformly in the early-to-mid thirties. Among comparative studies, the mean ages were nearly identical: Lin et al. (2024) reported 32.5 vs. 31.8 years (*p* = 0.061) [25], and Hessami et al. (2019) reported 30.5 vs. 31.8 years for MC and DC groups [26], respectively. The DC-only cohort showed a similar range, from 29.6 years (Yüce et al., 2016) to 36.8 years (Geisler et al., 2014) [27,28].

##### BMI

BMI was reported less consistently (in 18 of 35 studies) and demonstrated substantially greater variability, particularly in the DC cohort. Among comparative studies reporting BMI, the values were comparable between the chorionicity: Sun et al. (2016) reported 22.2 vs. 22.5 kg/m^2^ [29], and Hessami et al. (2019) reported 26.0 vs. 26.4 kg/m^2^ [26]. However, the absolute values differed markedly between these two studies, reflecting different population baselines (Chinese vs. Iranian cohort). The DC cohort exhibited the widest variation, with mean BMIs ranging from 21.6 kg/m^2^ (Seravalli et al., 2020, for an Italian cohort [30]) to 29.3 kg/m^2^ (Dai et al., 2022, for a Chinese cohort [31]).

##### Other Demographics

Nulliparity was consistently highly prevalent among ART-conceived twin pregnancies irrespective of the chorionicity, with comparative studies demonstrating nearly identical rates between MC and DC pregnancies, including 92.1% versus 92.8% in the large cohort by Lin et al. (2024) [25], 84% vs. 86.9% and 90.0% versus 94.5% in the studies by Simões et al. (2015) [32], and Sun et al. (2016) [29]. Among the DC-only cohort, nulliparity ranged from 55.0% to 90.3%. Smoking prevalence was generally low in the studies reporting these data, ranging from 1.1% to 20.0%. Prior cesarean delivery was relatively uncommon overall, reported in 4.7% [28] to 16.7% [39] of the DC cohort. The reported duration of infertility appeared to be similar between the chorionicity groups where available, with Lin et al. (2024) reporting mean durations of 3.43 years in MC pregnancies, and 3.04 years in DC pregnancies [25], while Zhou et al. (2016) reported a mean duration of 4.20 years in a large DC cohort [33].

#### 3.3.2. Obstetrical Outcomes

##### Gestational Age and Preterm Birth

MC ART twins were consistently delivered 1–2 weeks earlier than DC ART twins, with this difference most pronounced in larger, higher-quality studies. Among comparative studies, Simões et al. (2015) reported a significantly lower mean gestational age in MC vs. DC pregnancies (33.1 vs. 35.5 weeks; *p* = 0.004) [32], a difference of 2.4 weeks. Sun et al. (2016) documented preterm birth < 32 weeks in 27.6% of MC vs. 5.3% of DC pregnancies, and delivery < 28 weeks in 17.2% vs. 1.0%, highlighting the vulnerability of MC pregnancies to extreme prematurity [29]. The MC-only studies showed remarkable consistency, with mean gestational ages tightly clustered between 33.9 and 34.0 weeks across three studies (Roero et al., 2023 [34]; Van Lierde et al., 2022 [35]). By striking contrast, the DC-only studies demonstrated substantial heterogeneity, with mean gestational ages ranging from 33.6 weeks (Weghofer et al., 2009 [36]) to 37.0 weeks (Valenzuela-Alcaraz et al., 2018 [37]) (Table 2).

##### Hypertensive Disorders of Pregnancy

For gestational hypertension, comparative studies showed inconsistent patterns: Simões et al. (2015) found a non-significant trend toward higher rates in DC pregnancies (22.2% vs. 16.0%; OR = 1.6, 95% CI [0.7, 3.9]) [32]; Hessami et al. (2019) observed 14.3% in MC vs. 30.9% in DC [26]; Sun et al. (2016) found nearly identical rates (6.9% vs. 7.1%) [29]; and Trojner Bregar et al. (2016) reported no cases in MC vs. 7.5% in DC [38]. Preeclampsia rates showed similar inconsistency: Sun et al. (2016) reported 6.9% in MC vs. 10.5% in DC [29]; Sarais et al. (2015) reported 20.0% in their small MC cohort (n = 10) [23]. The DC-only studies exhibited extreme variation in preeclampsia rates, from 3.3% (Seravalli et al., 2020 [30]) to 40% (Valenzuela-Alcaraz et al., 2018 [37]), with most studies reporting rates between 7% and 14%. The extreme heterogeneity in the reported rates cannot be explained by chorionicity alone (Table 3).

##### GDM

The GDM rates varied dramatically across the studies without any consistent pattern by chorionicity. The variation likely reflects the differences in diagnostic criteria, population BMI, and screening practices. Among comparative studies, Sun et al. (2016) reported 10.3% in MC vs. 15.5% in DC pregnancies [29]; Simões et al. (2015) observed comparable rates (12.0% vs. 15.9%; OR = 0.7) [32]; Roero et al. (2023) found the highest MC rate at 24.4% [34]; Hessami et al. (2019) reported 14.3% in MC vs. 9.9% in DC [26]; and Trojner Bregar et al. (2016) observed 4.4% in MC and 6.1% in DC pregnancies [38]. Lin et al. (2024) reported GDM in 5.5% of MC pregnancies following frozen–thawed embryo transfer, compared to 9.2% in their DC cohort (OR = 0.66, 95% CI [0.28–1.35]) [25]. Among the DC-only studies, the GDM rates ranged dramatically from 3.7% (Fan et al., 2013 [49]) to 30.6% (Tang et al., 2025 [50]), with most studies reporting rates between 5% and 15%, though several exceeded 20% (Seravalli et al., 2020 [30]: 21.8%; Lin et al., 2021: 22.9% [58]) (Table 3).

##### CD

The CD rates were universally high across all ART twin pregnancies regardless of chorionicity, with most studies reporting rates exceeding 70%. Among comparative studies, Simões et al. (2015) reported comparable elective CD rates between MC (56.0%) and DC (50.9%) pregnancies (OR = 1.2; 95% CI [0.5, 2.9]), with similar unplanned CD rates (24% vs. 18.8%) [32]. Trojner Bregar et al. (2016) found comparable elective CD rates (44.4% vs. 31.4%), but unplanned CD was significantly higher in DC pregnancies (OR = 2.5, 95% CI [1.1, 5.7]) [38]. Lin et al. (2024) reported the highest rates overall, with CD in 97.6% of MC and 96.3% of DC pregnancies, likely reflecting institutional policy at a single large Chinese center rather than patient characteristics alone [25]. The DC-only studies confirmed this pattern of high rates, ranging from 62.7% to 98.8%, with the highest reported by Lin et al. (2021) (98.8%) [58], and Tang et al. (2025) (98.3%) [50] (Table 3).

##### Other Obstetrical Complications

Several less common outcomes were reported too inconsistently for meaningful synthesis. Placental abruption/previa showed no clear pattern by chorionicity: Hessami et al. (2019) reported a higher combined rate in MC (9.5%) than DC (3.9%) [26], while Sun et al. (2016) reported a lower rate in MC (10.3%) than DC (7.1%) [29], and Lin et al. (2024) found a higher rate in MC (2.4%) than DC (1.0%) [25]. The highest rates occurred in selected DC populations: Dai et al. (2022) reported 8.5% [31] and Romanski et al. (2018) reported 7.2% [45], both exceeding rates seen in most MC cohorts. Premature rupture of membranes showed inconsistent differences by chorionicity, with DC rates ranging from 3.7% to 23.8%. Cholestasis, postpartum hemorrhage, oligohydramnios, and polyhydramnios were too infrequently reported to allow for a meaningful comparison (Table 4).

#### 3.3.3. Neonatal Outcomes

##### Birth Weight and Low Birth Weight

MC twins had consistently lower birth weights than DC twins, with the difference most pronounced in studies that also reported the largest gestational age differences. However, low birth weight rates are heavily confounded by the gestational age at delivery: when the gestational age is similar between groups, the birth weight differences between the chorionicity appears to narrow or disappear. Among comparative studies, Simões et al. (2015) reported the largest difference: mean birth weight of 1754 g in MC vs. 2289 g in DC (*p* < 0.001), a gap of 535 g, which coincided with the largest gestational age difference in the literature (33.1 vs. 35.5 weeks), suggesting that much of the birth weight disparity is mediated by earlier delivery in MC pregnancies [32]. Lin et al. (2024), in the largest comparative cohort, found LBW in 70.3% of MC neonates vs. 54.0% of DC neonates (OR = 1.92, 95% CI [1.27–2.93]), confirming a higher LBW risk in MC pregnancies [25]. Trojner Bregar et al. (2016) found comparable mean birth weights (2282 g vs. 2274 g) and LBW rates (55.5% vs. 51.0%) when the gestational ages were similar (35.7 vs. 35.2 weeks) [38]. The MC-only studies showed mean birth weights consistently between 1750 and 2200 g. The DC-only studies exhibited extreme heterogeneity in the LBW rates, from 15.5% (Mohammed and Abdel-Maaboud, 2012 [51]) to 65.6% (Shlush et al., 2024 [39]), with this variation correlating strongly with the reported mean gestational ages (Table 5).

##### Apgar Score

Findings regarding the Apgar scores were inconsistent across the studies. Simões et al. (2015) reported no low 5-min Apgar scores (<7) in MC neonates (0/50) versus 1.4% (9/640) in DC neonates (*p* = 0.5) [32]. By contrast, Hessami et al. (2019) reported markedly higher rates in MC (23.8%, 10/42) than DC (10.2%, 37/362) [26]. That study also noted lower Apgar scores in first twins compared to second twins in both chorionicity groups. Fan et al. (2013) similarly observed higher low-Apgar rates in second-born twins (8.1% vs. 2.5%) within their DC cohort [49]. Among other DC-only studies, the rates ranged from 1.2% (Seravalli et al., 2020) to approximately 9% (Yang et al. (2011) [30,43].

##### Perinatal Mortality

Perinatal mortality was consistently higher in MC compared to DC ART twins, with the difference driven primarily by higher rates of intrauterine fetal demise and neonatal death in MC pregnancies. Among comparative studies, Simões et al. (2015) reported early neonatal mortality in 8.0% of MC vs. 0.9% of DC neonates (OR = 9.0, 95% CI [2.2–34.2]) [32]. Hessami et al. (2019) found IUFD more than twice as high in MC (19.0% vs. 7.2%), with overall perinatal mortality substantially higher in MC (23.8% vs. 14.4%) [26]. Sun et al. (2016) documented IUFD/stillbirth in 3.4% of MC vs. 0.5% of DC neonates [29]. Lin et al. (2024), in the largest cohort, reported stillbirth in 1.22% of MC vs. 0.08% of DC pregnancies (OR = 12.0, 95% CI [0.55–99.4]) and neonatal death in 2.47% vs. 0.8% (OR = 4.95, 95% CI [1.41–13.2]) [25]. While the confidence intervals are wide due to low event rates, the consistent direction of effect across all comparative studies is notable. Trojner Bregar et al. (2016) found no IUFD in MC vs. 1.3% in DC, with early neonatal death in 2.2% of MC vs. 1.7% of DC [38]. The DC-only mortality rates were generally low (<2%) but varied, with Moini et al. (2012) reporting a notably high neonatal mortality of 7.0% (32/460 neonates) [53]. Across all comparative studies, MC ART twins consistently demonstrated a 2- to 10-fold higher risk of perinatal mortality compared to DC ART twins.

##### NICU Admission

The NICU admission rates varied directly with the preterm birth rates and showed no independent association with chorionicity. Among comparative studies, Sun et al. (2016) reported the most dramatic disparity: 48.3% of MC vs. 0.7% of DC neonates required NICU care, reflecting their markedly different preterm birth rates (34.5% vs. 7.1% for <32 weeks) [29]. By contrast, Hessami et al. (2019) found comparable NICU rates (40.5% vs. 39.0%), consistent with their similar gestational age profiles [26]. The MC-only NICU admission rates ranged from 37.8% (Roero et al., 2023 [34]) to 55.0% (Sarais et al., 2015 [23]). The DC rates showed extreme variation, from 15% (Valenzuela-Alcaraz et al., 2018 [37]) to 50.8% (Yang et al., 2011 [43]); studies with the highest NICU admission rates (>45%) all reported preterm birth rates exceeding 40% (Yang et al., 2011 [43]; Duy Anh et al., 2022 [46]; Weghofer et al., 2009 [36]; Shlush et al., 2024 [39]).

##### RDS

The RDS rates followed the same pattern as NICU admission, varying directly with preterm birth rates. Among MC pregnancies, Roero et al. (2023) reported RDS in 23.3% [34], while Lin et al. (2024) found no cases, a disparity explained by different gestational age distributions across their respective cohorts [25]. The DC RDS rates ranged from 2.5% (Fan et al., 2013) [49], to 26.1% (Moini et al., 2012) [53], with the highest rates corresponding to studies with the lowest mean gestational ages (Moini et al., 2012: mean 34.5 weeks [53]; Geisler et al., 2014: 97.7% preterm birth rate [28]). Table 6 summarizes the neonatal morbidities.

## 4. Discussion

This systematic review provides evidence that MC twin pregnancies conceived through the use of ART carry a substantially greater burden of adverse perinatal outcomes than their DC counterparts, particularly regarding earlier gestational age at delivery, prematurity, low birth weight, and perinatal mortality. MC twins were delivered approximately one to two weeks earlier than DC twins across the comparative cohorts, and this earlier delivery appears to cascade into the observed deficits in birth weight and the elevated rates of low birth weight [29,32].

The mechanistic basis for the inferior neonatal outcomes observed in MC gestations is well grounded in placental pathophysiology. Unlike dichorionic twins, who possess separate placental masses with independent circulations, MC twins share a single placenta in which bidirectional vascular anastomoses (arterio-arterial, veno-venous, and arterio-venous) create hemodynamic interdependence between the two fetuses. These anastomoses are the substrate for twin-to-twin transfusion syndrome (TTTS), a condition characterized by net unidirectional blood transfer from a donor twin to a recipient twin, culminating in a progressive oligohydramnios–polyhydramnios sequence and, if untreated, fetal loss or severe neurological injury. Beyond TTTS, selective fetal growth restriction arising from unequal placental sharing can precipitate fetal hypoxia and impaired neurodevelopment in the growth-restricted co-twin, while acute hemodynamic shifts at the time of one twin’s in utero demise may transmit injurious pressure waves through anastomoses to the surviving twin [59,60]. These mechanisms collectively explain the higher rates of intrauterine fetal demise, stillbirth, and neonatal death in MC twins [61,62]. Importantly, these pathophysiological mechanisms operate independently of how the pregnancy was conceived; ART does not alter the vascular architecture of a monochorionic placenta, and the risks inherent to MC twinning are therefore not mitigated by the mode of conception [63,64,65].

In our systematic review, monochorionic twins consistently had lower birth weights than dichorionic twins, a finding that aligns with prior systematic reviews examining weight discordance and selective fetal growth restriction in twin pregnancies. D’Antonio et al. reported that birth weight discordance ≥ 25% was associated with a 4.7-fold higher risk of neonatal death in monochorionic twins without TTTS [66]. Groene et al. demonstrated that monochorionic twins with selective fetal growth restriction or birth weight discordance are at an increased risk for cerebral palsy and lower developmental test scores, with a within-pair disadvantage for the smaller twin [67]. These findings reinforce that chorionicity-driven complications, including weight discordance, perinatal mortality, and neurodevelopmental harm, operate independently of the conception mode, supporting our conclusion that ART-conceived MC twins require the same heightened surveillance as spontaneously conceived MC twins.

The pattern of outcomes documented here is consistent with, and extends, the findings from the broader literature on spontaneously conceived twin pregnancies. Large population-based studies of naturally conceived twins have established that monochorionicity independently predicts prematurity, perinatal mortality, and neurodevelopmental morbidity when compared with dichorionicity [61,62]. The retrospective cohort study by Hack et al. [68] demonstrated that, irrespective of the conception mode, MC twins exhibited higher rates of TTTS and intrauterine growth restriction than DC twins, reinforcing the primacy of chorionicity as the dominant determinant of certain perinatal risks. The systematic review by Marleen et al. (2021) [69] similarly reported that IVF-conceived MC twins were approximately 1.5 times more likely than their DC counterparts to deliver before 34 weeks of gestation, a threshold associated with substantially elevated neonatal morbidity and resource utilization. The present review corroborates these estimates and extends the evidence base to a broader range of ART modalities and clinical settings.

Much of the existing ART outcome literature has examined the comparative risks of ART versus spontaneous conception without stratifying by chorionicity, thereby obscuring the differential risk profile of MC twin pregnancies. For instance, a systematic review by Marleen et al. (2024) found that IVF twins, irrespective of chorionicity, had a higher risk of adverse outcomes but did not explore chorionicity-specific effects [70], leaving clinicians without the chorionicity-specific risk estimates needed to counsel and manage individual patients appropriately. The present review addresses this gap and represents, to our knowledge, one of the few systematic syntheses to focus specifically on chorionicity-stratified outcomes within the ART-conceived twin population. However, the gap is not yet fully closed: the relative scarcity of primary studies reporting the granular MC- versus DC-stratified data, combined with the marked numerical disparity between MC and DC pregnancies in the included cohorts means that precise effect estimates for several outcome categories, particularly rarer maternal complications, remain elusive and should be interpreted with appropriate caution.

In contrast to the relative consistency observed for neonatal outcomes, maternal complications, including hypertensive disorders, preeclampsia, GDM, PROM, and CD, demonstrated marked variability across the studies without a reproducible association with chorionicity. Several explanations are plausible. First, maternal complications in twin pregnancies are multifactorial, influenced by uterine volume, placental mass and implantation characteristics, endocrine milieu, and pre-existing maternal comorbidities; chorionicity may have a less direct or less uniform impact on these processes than on feto-placental hemodynamics. Second, diagnostic thresholds and classification schemes for conditions such as GDM and hypertensive disorders vary between national guidelines and have evolved substantially over the study period, introducing definitional heterogeneity that complicates cross-study comparisons. Third, the obstetric management of twin pregnancies, including the timing and mode of delivery, is influenced by local protocols and clinical judgment, which may differentially affect the measured prevalence of complications, such as cesarean delivery and PPROM, across healthcare systems. These factors collectively reduce the likelihood of detecting a reproducible chorionicity signal for maternal outcomes in the absence of large, prospectively designed studies with uniform outcome definitions.

The substantial clinical and statistical heterogeneity across the included studies warrants explicit discussion, as it has direct implications for both the interpretation of current findings and the design of future research. First, ART protocol variation was considerable: the included studies encompassed conventional in vitro fertilization, ICSI, fresh and frozen embryo transfer cycles, and varying practices regarding the number and developmental stage of the embryos transferred [25,39,41,47,58]. Such protocol diversity is well-recognized in the literature as a major source of between-study heterogeneity in ART outcome research [65,71,72]. Each of these variables may independently influence implantation biology, placental development, and downstream obstetric outcomes, making the disaggregation of chorionicity-specific effects from ART-specific effects methodologically challenging. Second, geographic and healthcare system diversity meant that antenatal surveillance schedules, thresholds for intervention in suspected TTTS or fetal growth restriction, neonatal intensive care capacity, and definitions of perinatal mortality were not standardized across the included populations. Third, the studies spanned more than two decades, a period that has witnessed substantial advances in embryo culture media, vitrification techniques for cryopreservation, and neonatal intensive care practices. Temporal secular trends in outcome rates may have introduced additional variability that is difficult to separate from chorionicity effects through a retrospective analysis alone. These sources of heterogeneity collectively precluded meaningful quantitative pooling via meta-analysis, and should be understood as a fundamental feature of the current evidence base rather than merely a statistical nuisance.

Several methodological limitations of this review merit acknowledgment. A majority of the included studies employed retrospective cohort designs, which are susceptible to selection bias, incomplete confounder adjustment, and missing data, particularly for outcomes that are not routinely documented in clinical records. Inconsistent definitions of key exposure and outcome variables, such as, for example, the gestational age thresholds used to define prematurity, the ultrasound criteria used to diagnose fetal growth restriction, and the biochemical or clinical criteria applied to diagnose GDM, limit comparability across the studies and likely contributed to the observed heterogeneity. The pronounced numerical imbalance between MC and DC pregnancies across all included cohorts, with MC twins comprising only a small fraction of the total study population, reduced the statistical precision for the MC-specific estimates and may have introduced unequal group sizes that affect the reliability of comparative analyses. Furthermore, while this review was designed to include a meta-analysis, the degree of clinical and methodological diversity encountered rendered quantitative pooling inappropriate, limiting the ability to generate summary effect estimates.

Despite these limitations, the findings of this review carry meaningful clinical implications for ART counseling, antenatal surveillance, and obstetric management. In the context of patient counseling, clinicians should communicate to couples undergoing ART that, while a multiple embryo transfer increases the probability of twin conception, the rare but consequential occurrence of MC twinning confers a risk profile that is qualitatively different from and substantially higher than that of DC twins. This distinction should be explicitly incorporated into pre-treatment informed consent discussions, particularly given that elective single-embryo transfer (eSET) policies, already widely adopted to reduce overall twin rates, also indirectly reduce the absolute incidence of MC twinning. Couples who conceive MC twins through the use of ART should be counseled that the subsequent pregnancy trajectory is governed primarily by placental biology rather than by their mode of conception, and that close specialist follow-up is essential. Regarding antenatal surveillance, current international guidelines recommend fortnightly sonographic monitoring of MC twin pregnancies from 16 weeks of gestation to detect TTTS, selective growth restriction, and other complications at a stage when intervention may improve outcomes. The present findings affirm the appropriateness of this approach within the ART context, and suggest that surveillance protocols developed for spontaneously conceived MC twins should be applied equally to ART-conceived MC pregnancies. From a management perspective, the consistently earlier gestational age at delivery among MC twins observed in this review underscores the importance of individualized delivery timing decisions, balancing the risks of continued in utero exposure against those of iatrogenic prematurity, ideally within a multidisciplinary framework that includes maternal–fetal medicine specialists and experienced neonatologists.

The evidence gaps identified in this review point to several priorities for future research. Prospective, multicenter studies with pre-specified, standardized outcome definitions are urgently needed to generate reliable chorionicity-stratified effect estimates within ART-conceived twin populations. A larger cohort of MC twin pregnancies, potentially achieved through international consortium designs, is required to achieve adequate statistical power for rare outcome events and to enable meaningful subgroup analyses. Future investigations should also examine the influence of contemporary ART practices, including the widespread adoption of preimplantation genetic testing and the global shift toward frozen embryo transfer cycles, on the incidence of embryo splitting, and the subsequent obstetric profile of MC twins. Finally, the development and prospective validation of evidence-based surveillance and management protocols specifically tailored to ART-conceived MC twin pregnancies would represent a meaningful step toward optimizing outcomes in this high-risk group and improving the quality of patient counseling at the outset of treatment.

## 5. Conclusions

ART-conceived monochorionic twin pregnancies are associated with higher risks of prematurity, low birth weight, and perinatal mortality compared with dichorionic pregnancies, whereas maternal outcomes show no consistent relationship with chorionicity. These findings highlight the importance of chorionicity-specific surveillance and management in ART twin pregnancies. Further large-scale prospective studies with standardized chorionicity-specific outcome reporting are needed to better define the impact of chorionicity in ART-conceived twin pregnancies.

## Figures and Tables

**Table 1:** Characteristics Of the Included Studies. ART: Assisted Reproductive Technology, DC: Dichorionic Twin, GIFT: Gamete Intrafallopian Transfer, ICSI: Intracytoplasmic Sperm Injection, IVF: In Vitro Fertilization, MC: Monochorionic Twin.

Study (Year) [Ref]	Chorionicity		Country	Study Design	Pregnancies (n)	ART Protocol	Quality
**Monochorionic twins (MC) ** (Total n = 371)							
Simões et al. (2015) [32]		MC	Portugal	Retrospective cohort study	25	ART	Fair
Trojner Bregar et al. (2016) [38]		MC	Slovenia	Retrospective cohort study	45	ART	Moderate
Sun et al. (2016) [29]		MC	China	Retrospective cohort study	29	ART	Good
Roero et al. (2023) [34]		MC	Italy	Retrospective cohort study	45	IVF/ICSI	Good
Van Lierde et al. (2022) [35]		MC	Belgium	Retrospective cohort study	32	IVF/ICSI	Good
Hessami et al. (2019) [26]		MC	Iran	Retrospective cohort study	21	IVF/ICSI	Moderate
Lin et al. (2024) [25]		MC	China	Retrospective cohort study	164	IVF/ICSI	Moderate
Sarais et al. (2015) [23]		MC	Italy	Retrospective cohort study	10	IVF	Moderate
**Dichorionic twins (DC) ** (Total n = 15,277)							
Shlush et al. (2024) [39]		DC	Israel	Retrospective cohort study	389	IVF	Moderate
Vasario et al. (2010) [40]		DC	Italy	Prospective cohort study	84	IVF/ICSI	Moderate
Shavit et al. (2019) [41]		DC	Israel	Retrospective cohort study	773	ART	Moderate
Valenzuela-Alcaraz et al. (2018) [37]		DC	Spain	Prospective cohort study	50	IVF/ICSI	Moderate
Seravalli et al. (2020) [30]		DC	Italy	Retrospective cohort study	367	IVF/ICSI	Moderate
Weghofer et al. (2010) [42]		DC	Austria	Retrospective cohort study	113	IVF	Moderate
Yang et al. (2011) [43]		DC	South Korea	Retrospective cohort study	67	IVF	Moderate
Weghofer et al. (2009) [36]		DC	Austria	Retrospective cohort study	106	IVF	Good
Sun et al. (2016) [29]		DC	China	Retrospective cohort study	382	ART	Good
Barda et al. (2016) [44]		DC	Israel	Retrospective cohort study	449	IVF	Moderate
Romanski et al. (2018) [45]		DC	USA	Retrospective cohort study	291	IVF	Moderate
Duy Anh et al. (2022) [46]		DC	Vietnam	Single-center cohort study	483	IVF/ICSI	Moderate
Dai et al. (2022) [31]		DC	China	Retrospective cohort study	117	IVF	Moderate
Hessami et al. (2019) [26]		DC	Iran	Retrospective cohort study	181	IVF/ICSI	Moderate
Kuwata et al. (2004) [47]		DC	Japan	Retrospective cohort study	199	IVF/ICSI/GIFT	Moderate
Mor et al. (2020) [48]		DC	Israel	Retrospective cohort study	135	IVF	Good
Fan et al. (2013) [49]		DC	China	Retrospective cohort study	162	IVF/ICSI	Good
Tang et al. (2025) [50]		DC	China	Retrospective cohort study	2485	IVF	Moderate
Yüce et al. (2016) [27]		DC	Turkey	Retrospective cohort study	165	IVF	Moderate
Geisler et al. (2014) [28]		DC	Israel	Retrospective cohort study	171	IVF/ICSI	Moderate
Mohammed & Abdel-Maaboud (2012) [51]		DC	Qatar	Retrospective cohort study	145	IVF	Moderate
Pradhan et al. (2016) [52]		DC	India	Retrospective cohort study	135	ART	Moderate
Moini et al. (2012) [53]		DC	Iran	Prospective cohort study	230	IVF/ICSI	Moderate
Zhou et al. (2016) [33]		DC	China	Retrospective cohort study	662	IVF	Moderate
Atasoy Karakas et al. (2023) [54]		DC	Turkey	Retrospective cohort study	122	IVF/ICSI	Moderate
Haas et al. (2014) [55]		DC	Israel	Prospective cohort study	78	IVF	Moderate
Lin et al. (2024) [25]		DC	China	Retrospective cohort study	6101	IVF/ICSI	Moderate
Simões et al. (2015) [32]		DC	Portugal	Retrospective cohort study	320	IVF	Fair
Trojner Bregar et al. (2016) [38]		DC	Slovenia	Retrospective cohort study	776	ART	Moderate
Szymusik et al. (2012) [56]		DC	Poland	Retrospective cohort study	43	IVF	Moderate
Pinzauti et al. (2016) [57]		DC	Italy	Retrospective cohort study	430	ART	Good
Lin et al. (2020) [58]		DC	China	Retrospective cohort study	1084	IVF/ICSI	Moderate

**Table 2:** Maternal Demographics, Gestational Age, and Preterm Birth BMI = Body Mass Index; MC = Monochorionic; DC = Dichorionic; wks = weeks; N/R = Not Reported.

Study (Year) [Ref]	Pregnancies (n)	Maternal Age	BMI (kg/m^2^)	Gestational Age (wks)	Preterm Birth (n)
**Monochorionic twins (MC)**					
Simões et al. (2015) [32]	25	33.9 ± 5.4	N/R	33.1 ± 3.7	N/R
Trojner Bregar et al. (2016) [38]	45	32.1 ± 3.7	N/R	35.7 ± 2.5	N/R
Sun et al. (2016) [29]	29	32.9 ± 3.5	22.2 ± 3.9	N/R	N/R
Roero et al. (2023) [34]	45	36.7 ± 5.7	23.2 ± 4.7	33.9 ± 2	12
Van Lierde et al. (2022) [35]	32	33 ± 4	N/R	34 ± 4	N/R
Hessami et al. (2019) [26]	21	30.5 ± 4.6	26 ± 3.3	32.4 ± 4.2	8
Lin et al. (2024) [25]	164	32.5 ± 4.13	N/R	N/R	119
Sarais et al. (2015) [23]	10	35.2 ± 2.3	20.2 ± 1.8	N/R	2
**Dichorionic twins (DC)**					
Shlush et al. (2024) [39]	389	N/R	N/R	N/R	251
Vasario et al. (2010) [40]	84	33.5 ± 4.1	22.3 ± 3.8	34.9 ± 2.6	N/R
Shavit et al. (2019) [41]	773	N/R	N/R	N/R	320
Valenzuela-Alcaraz et al. (2018) [37]	50	35 ± 3	23 ± 4.2	37 ± 2.5	N/R
Seravalli et al. (2020) [30]	367	37 (34–42)	21.6 (20.1–24.3)	37 (34–42)	235
Weghofer et al. (2010) [42]	113	31.6 ± 4.2	23.7 ± 4.3	33.8 ± 2.9	106
Yang et al. (2011) [43]	67	32.5 ± 3.5	N/R	35.3 ± 2.8	18
Weghofer et al. (2009) [36]	106	31.6 ± 4.2	23.7 ± 4.3	33.6 ± 2.9	N/R
Sun et al. (2016) [29]	382	32.8 ± 3.5	22.5 ± 3.1	N/R	N/R
Barda et al. (2016) [44]	449	32.0 ± 5.5	24.3 ± 5.1	35.5 ± 2.9	265
Romanski et al. (2018) [45]	291	35.0 ± 3.8	(Categorical)	35.5 ± 2.7	178
Duy Anh et al. (2022) [46]	483	31.1 ± 5.0	26.1 ± 3.1	36.2 ± 2.4	348
Dai et al. (2022) [31]	117	30.96 ± 3.88	29.31 ± 3.69	35.27 ± 2.05	64
Hessami et al. (2019) [26]	181	31.8 ± 5.8	26.4 ± 2.3	33.9 ± 3.9	74
Kuwata et al. (2004) [47]	199	31.5–34.5	N/R	36.1–36.8	N/R
Mor et al. (2020) [48]	135	N/R	N/R	36.2 (34.2–37.8)	N/R
Fan et al. (2013) [49]	162	31.4 ± 3.9	N/R	35.1 ± 2.63	107
Tang et al. (2025) [50]	2485	31.51 ± 3.53	N/R	35.82 ± 2.19	N/R
Yüce et al. (2016) [27]	165	29.63 ± 5.22	N/R	34.14 ± 3.79	138
Geisler et al. (2014) [28]	171	36.81 ± 4.23	N/R	N/R	167
Mohammed and Abdel-Maaboud (2012) [51]	145	34.5 ± 4.1	22.3 ± 3.8	34.9 ± 2.6	66
Pradhan et al. (2016) [52]	135	N/R	N/R	N/R	95
Moini et al. (2012) [53]	230	30.6 ± 4.3	28.4 ± 3.9	34.5 ± 2.5	N/R
Zhou et al. (2016) [33]	662	31.42 ± 3.76	N/R	35.23 ± 2.24	354
Atasoy Karakas et al. (2023) [54]	122	N/R	N/R	N/R	105
Haas et al. (2014) [55]	78	31.7 ± 4.15	N/R	36.35 ± 2.02	23
Lin et al. (2024) [25]	6101	31.8 ± 3.69	N/R	N/R	3043
Simões et al. (2015) [32]	320	33.7 ± 4.1	N/R	35.5 ± 2.1	N/R
Trojner Bregar et al. (2016) [38]	776	32.6 ± 4.1	N/R	35.2 ± 3.2	N/R
Szymusik et al. (2012) [56]	43	31.4 ± 2.6	N/R	34.93 ± 2.94	29
Pinzauti et al. (2016) [57]	430	N/R	N/R	N/R	305
Lin et al. (2021) [58]	1084	31.8 ± 3.9	22.0 ± 3.3	35.9 ± 1.8	789

**Table 3:** Hypertensive Disorders, GDM, and Cesarean Delivery Rates. GHTN = Gestational Hypertension; GDM = Gestational Diabetes Mellitus; MC = Monochorionic; DC = Dichorionic; N/R = Not Reported.

Study (Year) [Ref]	Pregnancies (n)	GHTN (n)	Preeclampsia (n)	GDM (n)	C-Section (n)
**Monochorionic Twins (MC)**					
Simões et al. (2015) [32]	25	4	N/R	3	20
Trojner Bregar et al. (2016) [38]	45	0	N/R	2	27
Sun et al. (2016) [29]	29	2	2	3	N/R
Roero et al. (2023) [34]	45	8	N/R	11	39
Van Lierde et al. (2022) [35]	32	N/R	N/R	N/R	21
Hessami et al. (2019) [26]	21	3	N/R	3	N/R
Lin et al. (2024) [25]	164	12	N/R	9	160
Sarais et al. (2015) [23]	10	2		N/R	9
**Dichorionic Twins (DC)**					
Shlush et al. (2024) [39]	389	20	N/R	N/R	350
Vasario et al. (2010) [40]	84	14	N/R	10	66
Shavit et al. (2019) [41]	773	N/R	N/R	N/R	485
Valenzuela-Alcaraz et al. (2018) [37]	50	N/R	20	7	35
Seravalli et al. (2020) [30]	367	33	12	80	324
Yang et al. (2011) [43]	67	N/R	9	N/R	N/R
Sun et al. (2016) [29]	382	27	40	59	N/R
Barda et al. (2016) [44]	449	38		19	332
Romanski et al. (2018) [45]	291	75	N/R	14	213
Duy Anh et al. (2022) [46]	483	N/R	44	62	415
Dai et al. (2022) [31]	117	N/R	117	28	113
Hessami et al. (2019) [26]	181	56	N/R	18	N/R
Mor et al. (2020) [48]	135	28	8	29	113
Fan et al. (2013) [49]	162	N/R	32	6	134
Tang et al. (2025) [50]	2485	N/R	353	762	2442
Yüce et al. (2016) [27]	165	N/R	15	N/R	159
Geisler et al. (2014) [28]	171	37	N/R	8	130
Mohammed and Abdel-Maaboud (2012) [51]	145	N/R	27	N/R	N/R
Pradhan et al. (2016) [52]	135	N/R	N/R	N/R	92
Moini et al. (2012) [53]	230	30	N/R	21	214
Atasoy Karakas et al. (2023) [54]	122	10	N/R	26	N/R
Haas et al. (2014) [55]	78	7	N/R	11	N/R
Lin et al. (2024) [25]	6101	471	N/R	564	5874
Simões et al. (2015) [32]	320	71	N/R	51	223
Trojner Bregar et al. (2016) [38]	776	58	N/R	47	489
Szymusik et al. (2012) [56]	43	5	N/R	8	40
Pinzauti et al. (2016) [57]	430	44	N/R	72	413
Lin et al. (2021) [58]	1084	28	84	248	1071

**Table 4:** Other Obstetrical Complications. Only DC studies reporting these outcomes are listed; PROM = Premature Rupture of Membranes; PPROM = Preterm Premature Rupture of Membranes; PPH = Postpartum Hemorrhage; MC = Monochorionic; DC = Dichorionic; N/R = Not Reported.

Study (Year) [Ref]	Pregnancies (n)	PROM/PPROM (n)	Placenta Abruption/Previa (n)	Cholestasis (n)	PPH (n)	Oligohydramnios (n)
**Monochorionic Twins (MC)**						
Simões et al. (2015) [32]	25	4	N/R	N/R	N/R	N/R
Sun et al. (2016) [29]	29	8	3	1	6	N/R
Roero et al. (2023) [34]	45	9	N/R	3	N/R	N/R
Hessami et al. (2019) [26]	21	2	2	N/R	N/R	0
Lin et al. (2024) [25]	164	42	4	3	N/R	N/R
**Dichorionic Twins (DC)**						
Shlush et al. (2024) [39]	389	N/R	61	14	N/R	N/R
Vasario et al. (2010) [40]	84	20	2	9	5	N/R
Seravalli et al. (2020) [30]	367	N/R	N/R	30	N/R	4
Yang et al. (2011) [43]	67	4	3	N/R	N/R	N/R
Sun et al. (2016) [29]	382	62	27	27	47	N/R
Barda et al. (2016) [44]	449	N/R	14	3	7	N/R
Romanski et al. (2018) [45]	291	N/R	21	N/R	17	N/R
Duy Anh et al. (2022) [46]	483	94	N/R	N/R	28	6
Dai et al. (2022) [31]	117	N/R	10	13	20	5
Hessami et al. (2019) [26]	181	38	7	N/R	N/R	12
Mor et al. (2020) [48]	135	N/R	3	8	5	N/R
Fan et al. (2013) [49]	162	36	6	N/R	8	N/R
Tang et al. (2025) [50]	2485	N/R	42	416	126	N/R
Geisler et al. (2014) [28]	171	N/R	N/R	9	N/R	N/R
Mohammed and Abdel-Maaboud (2012) [51]	145	12	22	N/R	14	N/R
Pradhan et al. (2016) [52]	135	13	N/R	N/R	N/R	N/R
Moini et al. (2012) [53]	230	46	5	N/R	16	5
Atasoy Karakas et al. (2023) [54]	122	28	N/R	N/R	N/R	2
Lin et al. (2024) [25]	6101	925	64	58	N/R	N/R
Simões et al. (2015) [32]	320	28	N/R	N/R	N/R	N/R
Szymusik et al. (2012) [56]	43	9	N/R	5	N/R	N/R
Pinzauti et al. (2016) [57]	430	60	16	20	N/R	N/R
Lin et al. (2021) [58]	1084	129	28	25	N/R	N/R

**Table 5:** Birth Weight and Growth Restriction. IUGR = Intrauterine Growth Restriction; SGA = Small for Gestational Age; LBW = Low Birth Weight (<2500 g); VLBW = Very Low Birth Weight (<1500 g); MC = Monochorionic; DC = Dichorionic; SD = Standard Deviation; N/R = Not Reported.

Study (Year) [Ref]	Pregnancies (n)	Birth Weight (Mean ± SD or Median)	IUGR (n)	SGA (n)	LBW (n)	VLBW (n)
**Monochorionic Twins (MC)**						
Simões et al. (2015) [32]	25	1754 ± 591	N/R	N/R	21	17
Trojner Bregar et al. (2016) [38]	45	2282 ± 548	N/R	N/R	50	8
Sun et al. (2016) [29]	29	2169 ± 609	N/R	10	N/R	7
Roero et al. (2023) [34]	45	(T1) 2041 ± 425; (T2) 1995 ± 442	10	N/R	N/R	N/R
Van Lierde et al. (2022) [35]	32	Large: 2183 ± 670; Small: 1954 ± 671	7	N/R	N/R	N/R
Hessami et al. (2019) [26]	21	N/R	6	N/R	21	9
Lin et al. (2024) [25]	164	N/R	N/R	19	111	7
Sarais et al. (2015) [23]	10	N/R	N/R	2	18	N/R
**Dichorionic Twins (DC)**						
Shlush et al. (2024) [39]	389	N/R	N/R	38	510	86
Vasario et al. (2010) [40]	84	2235 ± 557	N/R	41	N/R	N/R
Shavit et al. (2019) [41]	773	N/R	N/R	209/1444	896/1444	124/1444
Valenzuela-Alcaraz et al. (2018) [37]	50	2493 ± 603	N/R	7	N/R	N/R
Seravalli et al. (2020) [30]	367	2290 (1990–2600)	58	143	N/R	N/R
Weghofer et al. (2010) [42]	113	(T1) 2053.6 ± 540.6; (T2) 1968.8 ± 561.3	N/R	N/R	N/R	N/R
Yang et al. (2011) [43]	67	2305 ± 590	N/R	20	79	13
Weghofer et al. (2009) [36]	106	(T1) 2011.3 ± 527.9; (T2) 1927.9 ± 549.2	N/R	N/R	N/R	N/R
Sun et al. (2016) [29]	382	2463 ± 463	N/R	5	N/R	8
Barda et al. (2016) [44]	449	2227 ± 563	77	N/R	N/R	N/R
Romanski et al. (2018) [45]	291	2539 ± 610	N/R	123	118	N/R
Duy Anh et al. (2022) [46]	483	2297.7 ± 437.5	368	N/R	578	63
Dai et al. (2022) [31]	117	2266.26 ± 498.66	35	N/R	N/R	11
Hessami et al. (2019) [26]	181	N/R	53	N/R	186	44
Kuwata et al. (2004) [47]	199	ICSI: 2368; IVF: 2353; GIFT: 2360	N/R	N/R	N/R	N/R
Mor et al. (2020) [48]	135	N/R	22	N/R	N/R	17
Fan et al. (2013) [49]	162	2295.7 ± 513.5	N/R	N/R	N/R	(47
Tang et al. (2025) [50]	2485	N/R	91	N/R	N/R	N/R
Yüce et al. (2016) [27]	165	2177 ± 512	N/R	N/R	N/R	N/R
Geisler et al. (2014) [28]	171	(T1) 2471.8 ± 557.9; (T2) 2451.6 ± 559.6	37	N/R	142	25
Mohammed and Abdel-Maaboud (2012) [51]	145	N/R	N/R	24	45	40
Pradhan et al. (2016) [52]	135	N/R	N/R	N/R	144	26
Moini et al. (2012) [53]	230	(T1) 2153 ± 741; (T2) 2010 ± 730	105	N/R	284	83
Zhou et al. (2016) [33]	662	2519.65 ± 454.8	N/R	N/R	493	38
Atasoy Karakas et al. (2023) [54]	122	N/R	N/R	57	N/R	N/R
Haas et al. (2014) [55]	78	2365 ± 435	7	N/R	N/R	N/R
Lin et al. (2024) [25]	6101	N/R	N/R	376	3267	216
Simões et al. (2015) [32]	320	2289 ± 454	N/R	N/R	380	41
Trojner Bregar et al. (2016) [38]	776	2274 ± 597	N/R	N/R	791	154
Szymusik et al. (2012) [56]	43	N/R	3	N/R	N/R	7
Pinzauti et al. (2016) [57]	430	N/R	84	N/R	N/R	N/R
Lin et al. (2021) [58]	1084	2353.9 ± 415.8	N/R	145	N/R	N/R

**Table 6:** Perinatal Mortality, Apgar Scores, and Neonatal Morbidities. IUFD = Intrauterine Fetal Demise; RDS = Respiratory Distress Syndrome; NICU = Neonatal Intensive Care Unit; MC = Monochorionic; DC = Dichorionic; N/R = Not Reported.

Study (Year) [Ref]	Pregnancies (n)	IUFD (n)	Stillbirth (n)	Neonatal Mortality (n)	5 min Apgar <7 (n)	RDS (n)	NICU Admission (n)	Congenital Anomalies (n)
**Monochorionic Twins (MC)**								
Simões et al. (2015) [32]	25	0	N/R	4	0	N/R	N/R	5
Trojner Bregar et al. (2016) [38]	45	0	N/R	1	3	N/R	N/R	0
Sun et al. (2016) [29]	29	2	N/R	N/R	N/R	N/R	28	N/R
Roero et al. (2023) [34]	45	0	N/R	1	5	21	34	5
Van Lierde et al. (2022) [35]	32	0	N/R	N/R	N/R	N/R	N/R	N/R
Hessami et al. (2019) [26]	21	8	N/R	2	6	N/R	17	1
Lin et al. (2024) [25]	164	N/R	2	4	N/R	0	N/R	5
Sarais et al. (2015) [23]	10	1	N/R	0	N/R	N/R	11	1
**Dichorionic Twins (DC)**								
Shlush et al. (2024) [39]	389	18	N/R	N/R	13	68	360	N/R
Vasario et al. (2010) [40]	84	N/R	N/R	2	N/R	17	51	19
Shavit et al. (2019) [41]	773	N/R	N/R	N/R	N/R	N/R	N/R	38
Valenzuela-Alcaraz et al. (2018) [37]	50	N/R	N/R	N/R	N/R	N/R	15	N/R
Seravalli et al. (2020) [30]	367	2	N/R	N/R	9	N/R	N/R	N/R
Weghofer et al. (2010) [42]	113	N/R	N/R	N/R	N/R	N/R	103	N/R
Yang et al. (2011) [43]	67	N/R	N/R	3	13	N/R	68	10
Weghofer et al. (2009) [36]	106	N/R	N/R	N/R	N/R	N/R	(T1) 49; (T2) 53	N/R
Sun et al. (2016) [29]	382	4	N/R	N/R	N/R	N/R	5	N/R
Barda et al. (2016) [44]	449	0	N/R	N/R	21	39	N/R	N/R
Duy Anh et al. (2022) [46]	483	N/R	N/R	10	N/R	N/R	495	15
Hessami et al. (2019) [26]	181	26	N/R	26	23	N/R	141	24
Kuwata et al. (2004) [47]	199	N/R	N/R	N/R	N/R	N/R	N/R	ICSI:11; IVF:11; GIFT:14
Mor et al. (2020) [48]	135	N/R	N/R	N/R	2	14	57	N/R
Fan et al. (2013) [49]	162	N/R	N/R	4	17	8	N/R	N/R
Tang et al. (2025) [50]	2485	N/R	N/R	N/R	N/R	269	1485	N/R
Yüce et al. (2016) [27]	165	6	N/R	N/R	N/R	N/R	N/R	N/R
Geisler et al. (2014) [28]	171	0	N/R	1	N/R	41	137	8
Mohammed and Abdel-Maaboud (2012) [51]	145	N/R	N/R	3	28	N/R	70	14
Pradhan et al. (2016) [52]	135	N/R	N/R	N/R	N/R	N/R	N/R	4
Moini et al. (2012) [53]	230	7	N/R	32	43	120	160	16
Zhou et al. (2016) [33]	662	N/R	N/R	3	N/R	N/R	N/R	7
Atasoy Karakas et al. (2023) [54]	122	N/R	N/R	N/R	N/R	N/R	77	N/R
Haas et al. (2014) [55]	78	4	N/R	N/R	N/R	N/R	N/R	N/R
Lin et al. (2024) [25]	6101	N/R	5	49	N/R	20	N/R	188
Simões et al. (2015) [32]	320	4	N/R	6	9	N/R	N/R	20
Trojner Bregar et al. (2016) [38]	776	21	N/R	27	104	N/R	N/R	37
Szymusik et al. (2012) [56]	43	1	N/R	3	N/R	N/R	13	4
Lin et al. (2021) [58]	1084	N/R	N/R	N/R	N/R	140	905	N/R

## Data Availability

The data supporting the findings of this study are available from the corresponding author upon reasonable request.

## Supplementary Materials

The following supporting information can be downloaded at: https://www.mdpi.com/article/10.3390/jcm15124761/s1, Table S1: PRISMA Checklist [22]; Table S2: Outcome definitions reported in the studies included in the review [23,25,26,29,32,35,38,39].

## Abbreviations

ART	Assisted Reproductive Technology
BMI	Body Mass Index
CD	Cesarean Delivery
CI	Confidence Interval
DC	Dichorionic
GDM	Gestational Diabetes Mellitus
GHTN	Gestational Hypertension
GIFT	Gamete Intrafallopian Transfer
HIE	Hypoxic Ischemic Encephalopathy
ICP	Intrahepatic Cholestasis of Pregnancy
ICSI	Intracytoplasmic Sperm Injection
IUFD	Intrauterine Fetal Demise
IUGR	Intrauterine Growth Restriction
IUI	Intrauterine Insemination
IVF	In Vitro Fertilization
IVH	Intraventricular Hemorrhage
LBW	Low Birth Weight
MC	Monochorionic
NEC	Necrotizing Enterocolitis
NICU	Neonatal Intensive Care Unit
NIH	National Institutes of Health
N/R	Not Reported
OHSS	Ovarian Hyperstimulation Syndrome
OR	Odds Ratio
OS	Ovarian Stimulation
PPH	Postpartum Hemorrhage
PRISMA	Preferred Reporting Items for Systematic Reviews and Meta-Analyses
PROM	Premature Rupture of Membranes
PROSPERO	International Prospective Register of Systematic Reviews
PROST	Pronuclear Stage Tubal Transfer
RDS	Respiratory Distress Syndrome
SD	Standard Deviation
SGA	Small for Gestational Age
TTTS	Twin-to-Twin Transfusion Syndrome
VLBW	Very Low Birth Weight
ZIFT	Zygote Intrafallopian Transfer

## References

[1] Fishel S. (2018). First in vitro fertilization baby—This is how it happened. Fertil. Steril..

[2] Steptoe P.C., Edwards R.G. (1978). Birth after the reimplantation of a human embryo. Lancet.

[3] Graham M.E., Jelin A., Hoon A.H., Wilms Floet A.M., Levey E., Graham E.M. (2023). Assisted reproductive technology: Short-and long-term outcomes. Dev. Med. Child Neurol..

[4] Nik Hazlina N.H., Norhayati M.N., Shaiful Bahari I., Nik Muhammad Arif N.A. (2022). Worldwide prevalence, risk factors and psychological impact of infertility among women: A systematic review and meta-analysis. BMJ Open.

[5] CDC (2026). FastStats—Infertility. https://www.cdc.gov/nchs/fastats/infertility.htm.

[6] Passet-Wittig J., Greil A.L. (2021). On estimating the prevalence of use of medically assisted reproduction in developed countries: A critical review of recent literature. Hum. Reprod. Open.

[7] Tierney K., Cai Y. (2019). Assisted reproductive technology use in the United States: A population assessment. Fertil. Steril..

[8] Goisis A., Håberg S.E., Hanevik H.I., Magnus M.C., Kravdal Ø. (2020). The demographics of assisted reproductive technology births in a Nordic country. Hum. Reprod..

[9] Jain M., Fang E., Singh M. (2025). Assisted reproductive technology (ART) techniques. StatPearls.

[10] Huang J.Y., Rosenwaks Z. (2014). Assisted reproductive techniques. Methods Mol. Biol..

[11] Boulet S.L., Kirby R.S., Reefhuis J., Zhang Y., Sunderam S., Cohen B., Bernson D., Copeland G., Bailey M.A., Jamieson D.J. (2016). Assisted reproductive technology and birth defects among liveborn infants in Florida, Massachusetts, and Michigan, 2000–2010. JAMA Pediatr..

[12] Wang C., Lv H., Ling X., Li H., Diao F., Dai J., Du J., Chen T., Xi Q., Zhao Y. (2021). Association of assisted reproductive technology, germline de novo mutations and congenital heart defects in a prospective birth cohort study. Cell Res..

[13] Kawwass J.F., Badell M.L. (2018). Maternal and fetal risk associated with assisted reproductive technology. Obstet. Gynecol..

[14] Rashid D., Alalaf S. (2020). Maternal and perinatal outcomes in twin pregnancies conceived spontaneously and by assisted reproductive techniques: Cross-sectional study. East. Mediterr. Health J..

[15] Jiang F., Gao J., He J., Tang Y., Cao Y., Wang X., Liu X., Wang T., Liu X., Sun J. (2021). Obstetric outcomes for twins from different conception methods–A multicenter cross-sectional study from China. Acta Obstet. Gynecol. Scand..

[16] Arian S.E., Erfani H., Yadav G.S., Clark S., Gibbons W.E., Shamshirsaz A.A. (2021). Neonatal and maternal outcomes among twin pregnancies stratified by mode of conception in the United States. Fertil. Steril..

[17] Gillet E., Martens E., Martens G., Cammu H. (2011). Prelabour Caesarean Section following IVF/ICSI in Older-Term Nulliparous Women: Too Precious to Push?. J. Pregnancy.

[18] Anbazhagan A., Hunter A., Breathnach F.M., Mcauliffe F.M., Geary M.P., Daly S., Higgins J.R., Morrison J.J., Burke G., Higgins S. (2014). Comparison of outcomes of twins conceived spontaneously and by artificial reproductive therapy. J. Matern.-Fetal Neonatal Med..

[19] Bhandari S., Agrawal P., Ganguly I., Singh A., Gupta N. (2016). Perinatal outcome in assisted reproductive pregnancies: Comparative analysis of reduced versus unreduced gestation. Int. J. Reprod. Med..

[20] Göçmen A., Güven Ş., Bağci S., Çekmez Y., Şanlıkan F. (2015). Comparison of maternal and fetal outcomes of IVF and spontaneously conceived twin pregnancies: Three year experience of a tertiary hospital. Int. J. Clin. Exp. Med..

[21] Derom C.A., Vlietinck R.F., Thiery E.W., Leroy F.O., Fryns J.-P., Derom R.M. (2006). The east flanders prospective twin survey (EFPTS). Twin Res. Hum. Genet..

[22] Page M.J., McKenzie J.E., Bossuyt P.M., Boutron I., Hoffmann T.C., Mulrow C.D., Shamseer L., Tetzlaff J.M., Akl E.A., Brennan S.E. (2021). The PRISMA 2020 statement: An updated guideline for reporting systematic reviews. Br. Med. J..

[23] Sarais V., Paffoni A., Baffero G.M., Parazzini F., Persico N., Somigliana E. (2016). Estimating the risk of monochorionic twins in IVF pregnancies from the perspective of a prenatal diagnosis unit. Twin Res. Hum. Genet..

[24] Gualdi-Russo E., Zaccagni L. (2026). The Newcastle–Ottawa Scale for Assessing the Quality of Studies in Systematic Reviews. Publications.

[25] Lin J., Zhang K., Wu F., Wang B., Chai W., Zhu Q., Huang J., Lin J. (2024). Maternal and perinatal risks for monozygotic twins conceived following frozen-thawed embryo transfer: A retrospective cohort study. J. Ovarian Res..

[26] Hessami K., Kasraeian M., Moghaddamizadeh Shoushtari S., Hessami A. (2019). Maternal and Neonatal Outcomes of Monochorionic and Dichorionic Twin Pregnancies Following Assisted Reproductive Technology in Southern Iranian Women. Shiraz E-Med. J..

[27] Yüce T., Seval M.M., Kalaeat E., Özmen B., Koç A. (2016). Çoğul gebeliklerin ikize indirgenmesi: Doğal sonuçları elde etmek her zaman mümkün müdür?. Cukurova Med. J..

[28] Geisler M.E., O’Mahony A., Meaney S., Waterstone J.J., O’Donoghue K. (2014). Obstetric and perinatal outcomes of twin pregnancies conceived following IVF/ICSI treatment compared with spontaneously conceived twin pregnancies. Eur. J. Obstet. Gynecol. Reprod. Biol..

[29] Sun L., Zou G., Wei X., Chen Y., Zhang J., Okun N., Duan T. (2016). Clinical outcomes after assisted reproductive technology in twin pregnancies: Chorionicity-based comparison. Sci. Rep..

[30] Seravalli V., Maoloni L., Pasquini L., Bolzonella S., Sisti G., Petraglia F., Di Tommaso M. (2020). The impact of assisted reproductive technology on prenatally diagnosed fetal growth restriction in dichorionic twin pregnancies. PLoS ONE.

[31] Dai F., Pan S., Lan Y., Tan H., Li J., Hua Y. (2022). Pregnancy outcomes and risk factors for preeclampsia in dichorionic twin pregnancies after in vitro fertilization: A five-year retrospective study. BMC Pregnancy Childbirth.

[32] Simões T., Queirós A., Marujo A.T., Valdoleiros S., Silva P., Blickstein I. (2015). Outcome of monochorionic twins conceived by assisted reproduction. Fertil. Steril..

[33] Zhou L., Gao X., Wu Y., Zhang Z. (2016). Analysis of pregnancy outcomes for survivors of the vanishing twin syndrome after in vitro fertilization and embryo transfer. Eur. J. Obstet. Gynecol. Reprod. Biol..

[34] Roero S., Arduino S., Arese A., Fea T., Ferrando I., Scaltrito G., Casula V., Ronco A., Bossotti C., Zizzo R. (2023). Retrospective comparison of monochorionic diamniotic twin pregnancies stratified by spontaneous or artificial conception. J. Perinat. Med..

[35] Van Lierde A., Delagrange H., Russo F.M., Van der Merwe J., Devlieger R., Lewi L. (2022). Are there differences between monochorionic twin placentas after spontaneous and assisted conception?. Placenta.

[36] Weghofer A., Klein K., Stammler-Safar M., Worda C., Barad D.H., Husslein P., Gleicher N. (2009). Can prematurity risk in twin pregnancies after in vitro fertilization be predicted? A retrospective study. Reprod. Biol. Endocrinol..

[37] Valenzuela-Alcaraz B., Cruz-Lemini M., Rodríguez-López M., Goncé A., García-Otero L., Ayuso H., Sitges M., Bijnens B., Balasch J., Gratacós E. (2018). Fetal cardiac remodeling in twin pregnancy conceived by assisted reproductive technology. Ultrasound Obstet. Gynecol..

[38] Trojner Bregar A., Blickstein I., Verdenik I., Lucovnik M., Tul N. (2016). Outcome of monochorionic-biamniotic twins conceived by assisted reproduction: A population-based study. J. Perinat. Med..

[39] Shlush E., Sarhan T., Aiob A., Tannus S., Mikhail S.M., Lowenstein L., Sgayer I. (2024). Obstetrical and Neonatal Outcomes of in vitro Fertilization Twins after Fresh Embryo Transfer versus Frozen-Thawed Embryo Transfer. Gynecol. Obstet. Investig..

[40] Vasario E., Borgarello V., Bossotti C., Libanori E., Biolcati M., Arduino S., Spinelli R., Delle Piane L., Revelli A., Todros T. (2010). IVF twins have similar obstetric and neonatal outcome as spontaneously conceived twins: A prospective follow-up study. Reprod. Biomed. Online.

[41] Shavit M., Miller N., Schreiber H., Asali A., Ravid D., Harlev A., Levitas E., Har-Vardi I., Berkovitz A. (2019). Twin pregnancies and perinatal outcomes: A comparison between fresh and frozen embryo transfer: A two-centre study. Reprod. Biomed. Online.

[42] Weghofer A., Klein K., Stammler-Safar M., Worda C., Barad D.H., Husslein P., Gleicher N. (2010). The impact of fetal gender on prematurity in dichorionic twin gestations after in vitro fertilization. Reprod. Biol. Endocrinol..

[43] Yang H., Choi Y.S., Nam K.H., Kwon J.Y., Park Y.W., Kim Y.H. (2011). Obstetric and perinatal outcomes of dichorionic twin pregnancies according to methods of conception: Spontaneous versus in-vitro fertilization. Twin Res. Hum. Genet..

[44] Barda G., Gluck O., Mizrachi Y., Bar J. (2017). A comparison of maternal and perinatal outcome between in vitro fertilization and spontaneous dichorionic-diamniotic twin pregnancies. J. Matern.-Fetal Neonatal Med..

[45] Romanski P.A., Carusi D.A., Farland L.V., Missmer S.A., Kaser D.J., Walsh B.W., Racowsky C., Brady P.C. (2018). Perinatal and peripartum outcomes in vanishing twin pregnancies achieved by in vitro fertilization. Obstet. Gynecol..

[46] Anh N.D., Ha N.T., Toan N.K., Dat D.T., Thuong P.H., Giang D.T., Duc T.A., Anh B.X., Ha N.M., Duc N.M. (2022). Obstetric and perinatal outcomes of dichorionic-diamniotic twin pregnancies conceived by IVF/ICSI compared with those conceived spontaneously. Clin. Ter..

[47] Kuwata T., Matsubara S., Ohkuchi A., Watanabe T., Izumi A., Honma Y., Yada Y., Shibahara H., Suzuki M. (2004). The risk of birth defects in dichorionic twins conceived by assisted reproductive technology. Twin Res. Hum. Genet..

[48] Mor N., Machtinger R., Yinon Y., Toussia-Cohen S., Amitai Komem D., Levin M., Sivan E., Meyer R. (2020). Outcome of two sequential singleton pregnancies and twin pregnancies among primiparous women at advanced age undergoing IVF. Arch. Gynecol. Obstet..

[49] Fan C., Sun Y., Yang J., Ye J., Wang S. (2013). Maternal and neonatal outcomes in dichorionic twin pregnancies following IVF treatment: A hospital-based comparative study. Int. J. Clin. Exp. Pathol..

[50] Tang W.Z., Cai Q.Y., Wang Y.X., Shao L.Z., Zhang X., Li Z.M., Tian H., Liu T.H., Chen Y., Wang L. (2025). Comparative influence of inappropriate gestational weight gain on pregnancy outcomes in IVF-conceived and spontaneously conceived twin pregnancies. Int. J. Gynecol. Obstet..

[51] Mohammed A.-B.F., Abdel-Maaboud M. (2012). Obstetric and neonatal outcomes of IVF versus spontaneously conceived dichorionic twins. Middle East Fertil. Soc. J..

[52] Pradhan S., Kamath M.S., Selliah H.Y., Thomas S., Chandy A., Aleyamma T.K. (2016). Comparison of perinatal outcomes of vanishing twin and twin pregnancies conceived following assisted reproductive technology: A retrospective analysis. Middle East Fertil. Soc. J..

[53] Moini A., Shiva M., Arabipoor A., Hosseini R., Chehrazi M., Sadeghi M. (2012). Obstetric and neonatal outcomes of twin pregnancies conceived by assisted reproductive technology compared with twin pregnancies conceived spontaneously: A prospective follow-up study. Eur. J. Obstet. Gynecol. Reprod. Biol..

[54] Atasoy Karakas L., Esin S., Sahin Uysal N., Tohma Y.A., Onalan G., Zeyneloglu H.B. (2023). Granulocyte colony-stimulating factor in assisted reproductive technology treatment does not increase the risk of adverse perinatal outcomes in twin pregnancies. J. Obstet. Gynaecol..

[55] Haas J., Hourvitz A., Dor J., Elizur S., Yinon Y., Barzilay E., Shulman A. (2014). Perinatal outcome of twin pregnancies after early transvaginal multifetal pregnancy reduction. Fertil. Steril..

[56] Szymusik I., Kosinska-Kaczynska K., Bomba-Opon D., Wielgos M. (2012). IVF versus spontaneous twin pregnancies—Which are at higher risk of complications?. J. Matern.-Fetal Neonatal Med..

[57] Pinzauti S., Ferrata C., Vannuccini S., Di Rienzo G., Severi F.M., Petraglia F., Di Tommaso M. (2016). Twin pregnancies after assisted reproductive technologies: The role of maternal age on pregnancy outcome. Eur. J. Obstet. Gynecol. Reprod. Biol..

[58] Lin D., Li P., Fan D., Chen G., Wu S., Ye S., Ma H., Rao J., Zhou Z., Zeng M. (2021). Association between IVF/ICSI treatment and preterm birth and major perinatal outcomes among dichorionic-diamnionic twin pregnancies: A seven-year retrospective cohort study. Acta Obstet. Gynecol. Scand..

[59] Klaritsch P. (2026). Complications in monochorionic twin pregnancies. J. Perinat. Med..

[60] Nikkels P.G.J., Hack K.E.A., van Gemert M.J.C. (2008). Pathology of twin placentas with special attention to monochorionic twin placentas. J. Clin. Pathol..

[61] Ferraz Liz C., Domingues S., Guedes A., Lopes L. (2022). The impact of chorionicity and assisted reproductive therapies in obstetric and neonatal outcomes. J. Matern. Fetal Neonatal Med..

[62] Kristiansen M.K., Joensen B., Ekelund C.K., Petersen O.B., Sandager P. (2015). with the Danish Fetal Medicine Study Group. Perinatal outcome after first-trimester risk assessment in monochorionic and dichorionic twin pregnancies: A population-based register study. BJOG Int. J. Obstet. Gynaecol..

[63] Wang M., Chai J. (2022). Comparison of outcomes of monochorionic twin pregnancies conceived by assisted reproductive technology vs. spontaneous conceptions: A systematic review and meta-analysis. Front. Pediatr..

[64] Martínez-Varea A., Martínez-Gómez M., Novillo B., Domenech J., Morales-Roselló J., Diago-Almela V. (2023). Perinatal Outcomes of Monochorionic Twin Pregnancies Conceived Naturally Versus through Assisted Reproductive Techniques. J. Clin. Med..

[65] de Villiers S., Slaghekke F., Middeldorp J.M., Klumper F.J., Walther F.J., Oepkes D., Lopriore E. (2012). Arterio-arterial vascular anastomoses in monochorionic twin placentas with and without twin anemia-polycythemia sequence. Placenta.

[66] D’Antonio F., Odibo A.O., Prefumo F., Khalil A., Buca D., Flacco M.E., Liberati M., Manzoli L., Acharya G. (2018). Weight discordance and perinatal mortality in twin pregnancy: Systematic review and meta-analysis. Ultrasound Obstet. Gynecol..

[67] Groene S.G., Tollenaar L.S.A., Oepkes D., Lopriore E., van Klink J.M.M. (2019). The Impact of Selective Fetal Growth Restriction or Birth Weight Discordance on Long-Term Neurodevelopment in Monochorionic Twins: A Systematic Literature Review. J. Clin. Med..

[68] Hack K.E.A., Vereycken M., Torrance H.L., Koopman-Esseboom C., Derks J.B. (2018). Perinatal outcome of monochorionic and dichorionic twins after spontaneous and assisted conception: A retrospective cohort study. Acta Obstet. Gynecol. Scand..

[69] Marleen S., Dias C., Nandasena R., MacGregor R., Allotey J., Aquilina J., Khalil A., Thangaratinam S. (2021). Association between chorionicity and preterm birth in twin pregnancies: A systematic review involving 29 864 twin pregnancies. BJOG Int. J. Obstet. Gynaecol..

[70] Marleen S., Kodithuwakku W., Nandasena R., Mohideen S., Allotey J., Fernández-García S., Gaetano-Gil A., Ruiz-Calvo G., Aquilina J., Khalil A. (2024). Maternal and perinatal outcomes in twin pregnancies following assisted reproduction: A systematic review and meta-analysis involving 802 462 pregnancies. Hum. Reprod. Update.

[71] Wang D.-D., Sun M.-L., Song X.-J., Liu K.-X., Chen X., Li D.-R., Wang Y., Sun M.-H., Yang R., Feng Z.-F. (2025). Assisted reproductive technology and reproductive, perinatal, and maternal outcomes: Evidence from an umbrella review of systematic reviews with meta-analyses of randomized controlled trials. BMC Med..

[72] Veeramani M., Balachandren N., Hong Y.H., Lee J., Corno A.F., Mavrelos D., Kastora S.L. (2024). Assisted reproduction and congenital malformations: A systematic review and meta-analysis. Congenit. Anom..

