# Supplementary material for: Obstetrical and Neonatal Outcomes in Twin Pregnancies Based on Chorionicity: A Systematic Review of ART-Conceived Monochorionic vs. Dichorionic Twins
Source: J Clin Med. 2026 Jun 18;15(12):4761. doi: 10.3390/jcm15124761 (PMC13301653; doi:10.3390/jcm15124761)
Supplement: Supplementary file 1 [file jcm-15-04761-s001.zip › jcm-4341865-supplementary Table S2 6.18.pdf]

Supplementary Table S2: Outcome definitions reported in the studies included in the review.

Study	Chorionicity	Preeclampsia Definition	Gestational Hypertension (GHTN)	GDM Definition	Preterm Birth (PTB) Threshold	LBW / VLBW / SGA / IUGR
Simoes et al. (2015) [32]	MC/DC	Not explicitly defined	Not explicitly defined	Not explicitly defined	<36 wks	LBW <2500g & VLBW <1500g
Bregar et al. (2016) [38]	MC/DC	Not explicitly defined	Not explicitly defined	Not explicitly defined	<37 wks	LBW <2500g & VLBW <1500g
Sun et al. (2016) [29]	MC/DC	BP ≥140/90 mmHg + Proteinuria (≥300 mg/24h or dipstick)	BP ≥140/90 mmHg without proteinuria	OGTT (unspecified load)	<32 wks (Very PTB), <28 wks	LBW & VLBW <1500g & SGA
Lierde et al. (2022) [35]	MC	Not reported	Not reported	Not reported	Not reported	Not explicitly defined/reported
Hessami et al. (2019) [26]	MC/DC	BP ≥140/90 mmHg + Proteinuria (≥300 mg/24h or dipstick)	BP ≥140/90 mmHg without proteinuria	100g OGTT	<37 wks	IUGR/FGR <10th percentile
Lin et al. (2024) [25]	MC/DC	Not explicitly defined	Not explicitly defined	Not explicitly defined	<37 wks, <32 wks (Very PTB)	LBW <2500g & VLBW <1500g & SGA <10th percentile
Sarais et al. (2015) [23]	MC	Not explicitly defined	Not explicitly defined	Not explicitly defined	<34 wks	LBW <2500g & SGA <10th percentile
Shlush et al. (2024) [39]	DC	Not explicitly defined	Not explicitly defined	Not reported	<37 wks	LBW <2500g & VLBW <1500g & SGA <10th percentile
